# Acute Pancreatitis with Pancreas Bifidum Mimicking Duodenitis on Computed Tomography

**DOI:** 10.5334/jbsr.3016

**Published:** 2023-02-13

**Authors:** Charles Deflandre, Romain Gillard

**Affiliations:** 1 CHU Sart-Tilman, BE

**Keywords:** pancreas bifidum, pancreatitis, MRI, fish-tail pancreas

## Abstract

**Teaching Point:** The bifid pancreas is an extremely rare congenital branching anomaly, knowledge of which is necessary in order to make the correct diagnosis in the event of associated pancreatitis.

## Case History

A 65-year-old patient presented to the emergency department with acute abdominal pain irradiated in the back for four days on a chronic background. There was no fever, nausea, or vomiting. The patient was known to have active hepatitis C (HC). The blood test showed signs of acute pancreatitis (AP) and inflammation.

**Figure 1:**
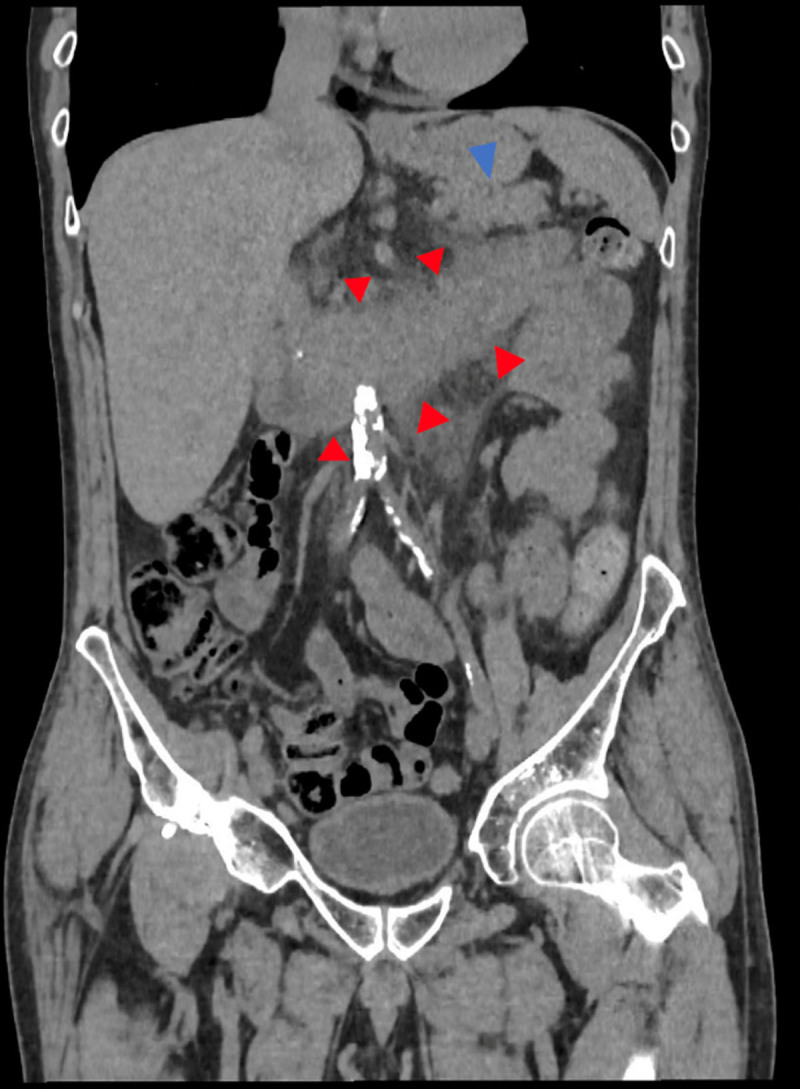


A computed tomography (CT) scan was performed without iodinated contrast media (ICM) due to an allergy. It showed thickening of the third and fourth duodenums with infiltration of the adjacent fat (Figure 1, red arrowheads). There was evidence of predominantly cephalic calcified chronic pancreatitis (CP) (not shown) with no acute component (Figure 1, blue arrowheads). At this stage the diagnosis of duodenitis was suspected.

Due to the discordance between biology and CT, the allergy to ICM, and the context of uncontrolled active HC, magnetic resonance imaging (MRI) was performed.

**Figure 2:**
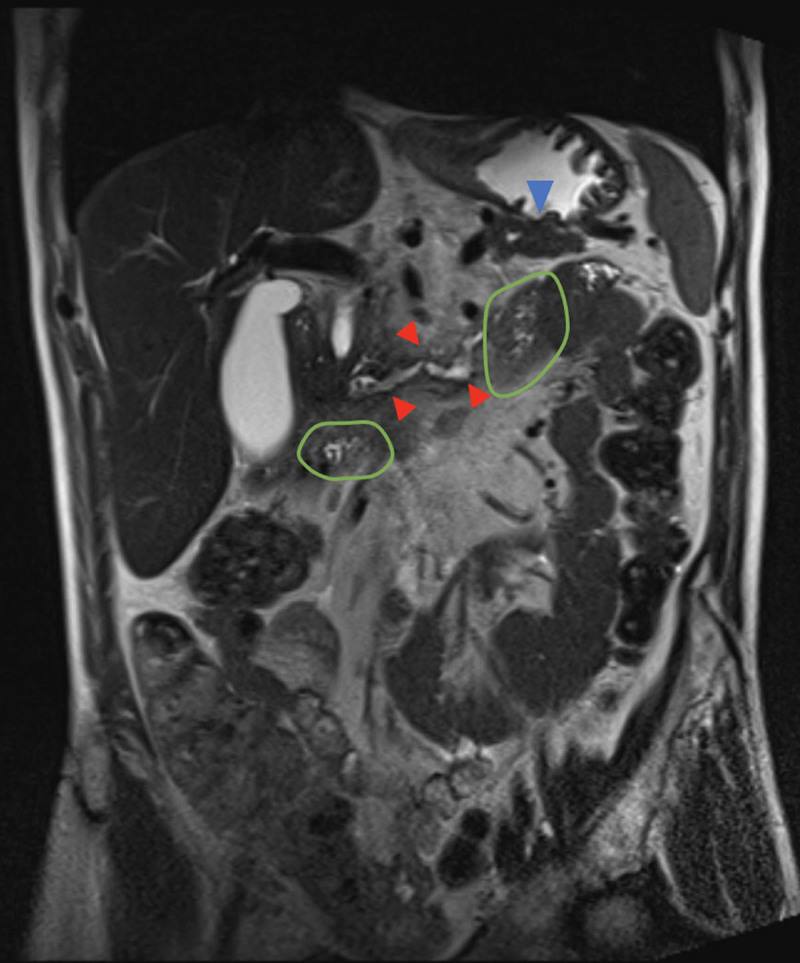


**Figure 3:**
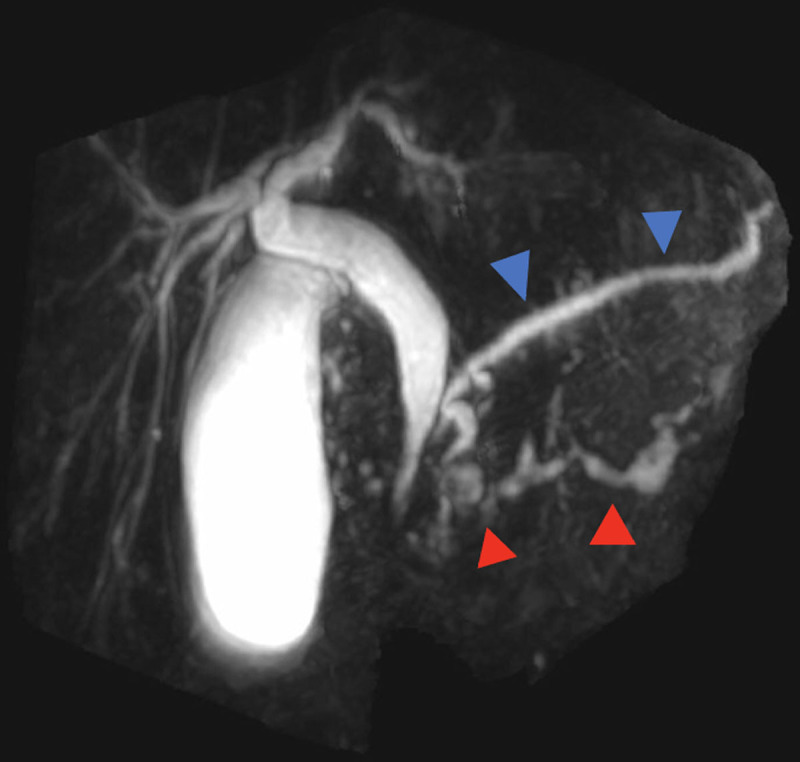


The MRI showed a duplication of the main pancreatic duct with two separate ducts that join at the pancreas head and draining via the major papilla. The diagnosis of pancreas bifidum (PB) was made. The ventral duct was eutopic (Figures 2 and 3, blue arrowheads). The dorsal duct was ectopic and ran along the third and fourth duodenums (Figures 2 and 3, red arrowheads. Green circles show the duodenum). Both ducts presented signs of CP especially the ventral duct with an atrophic appearance associated with multi-level ductal stenosis. There was an associated locoregional edema compatible with concomitant AP (not shown). A cholelithiasis cause was ruled out.

The patient showed a good clinical and biological evolution when provided with the adequate treatment.

## Comments

The embryological development of the pancreas is complex, PB is an extremely rare congenital branching anomaly whose prevalence is not known [1]. Only 25 cases are reported in the literature, and the vast majority concern only duplication of the tail of the pancreas [1]. Only three cases of AP on PB have been reported [1].

Usually the discovery is incidental and the patient asymptomatic. The association with AP is debated but seems to be the cause when the etiological research is negative, especially when there are signs of CP, as in our case [1]. No follow-up has been established for this congenital anomaly.

Our case is particularly unique. Indeed, the diagnosis could not be established with CT due to the topography of the dorsal branch making it impossible to distinguish it from the duodenum in spontaneous contrast. MRI was therefore decisive for the diagnosis of PB as well as the associated signs of AP and CP.
